# Computed Tomography and Magnetic Resonance Imaging in the Diagnosis of Cerebral Paragonimiasis in Children

**DOI:** 10.3389/fneur.2022.852334

**Published:** 2022-06-01

**Authors:** Jinhui Du, Likun Liu, Haiqing Fan, Yue Yu, Yilin Luo, Hui Yu, Xin Liao

**Affiliations:** Department of Medical Imaging, The Affiliated Hospital of Guizhou Medical University, Guiyang, China

**Keywords:** cerebral, paragonimiasis, children, computed tomography, magnetic resonance imaging

## Abstract

**Objective:**

To investigate the computed tomography (CT) and magnetic resonance image (MRI) manifestations of cerebral paragonimiasis (CP) in children and to improve the understanding of the disease.

**Methods:**

The cranial CT and MRI data of 12 children with positive intradermal tests for *Paragonimus*-specific antigens were retrospectively analyzed. Additionally, the lesion locations, morphology, and imaging characteristics were analyzed.

**Results:**

The lesions were located in the cerebral parenchyma in 12 cases, with 10 in the supratentorial area and two in the subtentorial area, among which three cases included involvement of the meninges. The morphology of the lesions was mainly nodular and striated, with clear or indistinct borders and varying degrees of surrounding edema. The lesions showed isodense or slightly hyperdense opacities on the CT scans, heterogeneous equal or slightly decreased signal intensities on the T1-weighted images (T_1_WI), heterogeneous equal or slightly increased signal intensities on the T2-weighted images (T_2_WI), and equal or slightly increased signal intensities on the diffusion-weighted images (DWI) in MRIs. In four cases, the cyst wall showed equal T1 and short T2 signals, and in six cases, the characteristic “tunnel sign” and “worm-eaten sign” were visible. The contrast-enhanced MRI showed strip-shaped enhancement in five cases, nodular or ring-shaped enhancement in three cases, linear enhancement in two cases, and uneven enhancement in two cases. The meninges adjacent to the lesions were thickened with significant enhancement in four cases.

**Conclusion:**

CP was mostly located in the cerebral parenchyma with involvement of the adjacent meninges. CT and MRI scans had certain imaging characteristics, and the MRI may particularly be of great value for the diagnosis of CP.

## Introduction

Cerebral paragonimiasis (CP) is a condition in which larvae of *Paragonimus* in the abdominal or thoracic cavity migrate upward from the mediastinum, travel up through the soft tissues surrounding the carotid artery, and enter the brain along bony holes such as the carotid canal and rupture foramen, forming an intracerebral abscess or granuloma. Clinically, the patient has a history of eating raw crayfish or stone crabs; they may have fever, convulsions, epilepsy, vomiting, and other symptoms, as well as exhibit subcutaneous migratory masses and test positive for *Paragonimus* (1, 2). Early diagnosis of CP is the key to achieving a good prognosis, thus proper imaging is particularly important to provide a reference for early diagnosis and to help clinicians develop the correct therapeutic plan (3, 4). Computed tomography (CT) and magnetic resonance imaging (MRI) facilitate the localization and qualitative diagnosis of lesions and are of great value for the diagnosis of CP (5). In the present study, the CT and MRI manifestations of 12 cases in our hospital with a clinically confirmed diagnosis of CP were retrospectively analyzed to improve the understanding of the disease. The diagnosis of CP was based on the application of an enzyme-linked immunosorbent assay (ELISA) with confirmation of serum antibodies to *Paragonimus* (4).

## Materials and Methods

### Clinical Data

The clinical data, along with CT and MRI imaging, were collected from 12 pediatric patients with CP, including 8 males and 4 females, aged 6–14 years, with a median age of 9 years old. All cases originated from Guizhou Province, China. Of the 12 patients, 9 had dizziness and headache, 6 had nausea and vomiting, 5 had limb movement or sensory disturbances, and 4 had seizures. Nine patients were treated with the insect repellents Praziquantel. Two patients were treated with oral insect repellents Praziquantel after lesion resection + decompressive craniectomy. One patient was discharged after intracranial decompression and went to another hospital for surgical treatment with unknown information concerning the drug administration and surgical therapy. The patients were followed up 1 year after discharge. There were no significant abnormal symptoms in eight patients, significant improvement of the symptoms in two patients, and the existence of limb weakness in one patient. While one patient was lost during the follow-up.

### Scanning Technique

The patients were scanned using a Siemens SOMATOM Sensation 16-row spiral CT scanner and a GE Signa 1.5T superconducting head-specific MRI scanner. Both plain CT and contrast-enhanced scanning were conducted with a thickness layer of 5 mm. The contrast enhancement was conducted with conventional scanning after an injection of 80 ml of non-ionic iodine contrast agent through the elbow vein. The parameters in MRI scanning were as follows: T1-weighted images (T_1_WI), T2-weighted images (T_2_WI), T2 fluid-attenuated inversion recovery (T_2_/FLAIR), and diffusion-weighted images (DWI) were scanned. The T_1_WI axial, coronal, and sagittal scans were conducted with an injection of gadopentetate glucosamine (Gd-DTPA) through the elbow vein at 0.1 mmol/kg of body weight at the time of enhancement.

## Results

### The Clinical Characteristics

The clinical manifestations in the 12 pediatric patients with paragonimiasis were mainly dizziness, headache, vomiting, seizure, and hemiparesis, with varying degrees of respiratory symptoms. With inquiry into the relevant clinical history, it was found that all 12 children had a history of eating raw stream crabs and drinking raw stream water, thus the clinical diagnosis should be highly suspicious of CP. The insect repellents Praziquantel is the preferred choice for the treatment of cerebral paragonimiasis. Praziquantel was administered and achieved good therapeutic effects in the remaining 11 patients except for one case who went to another hospital for treatment in the present group.

### The CT and MRI Manifestations

**Figure 1:**
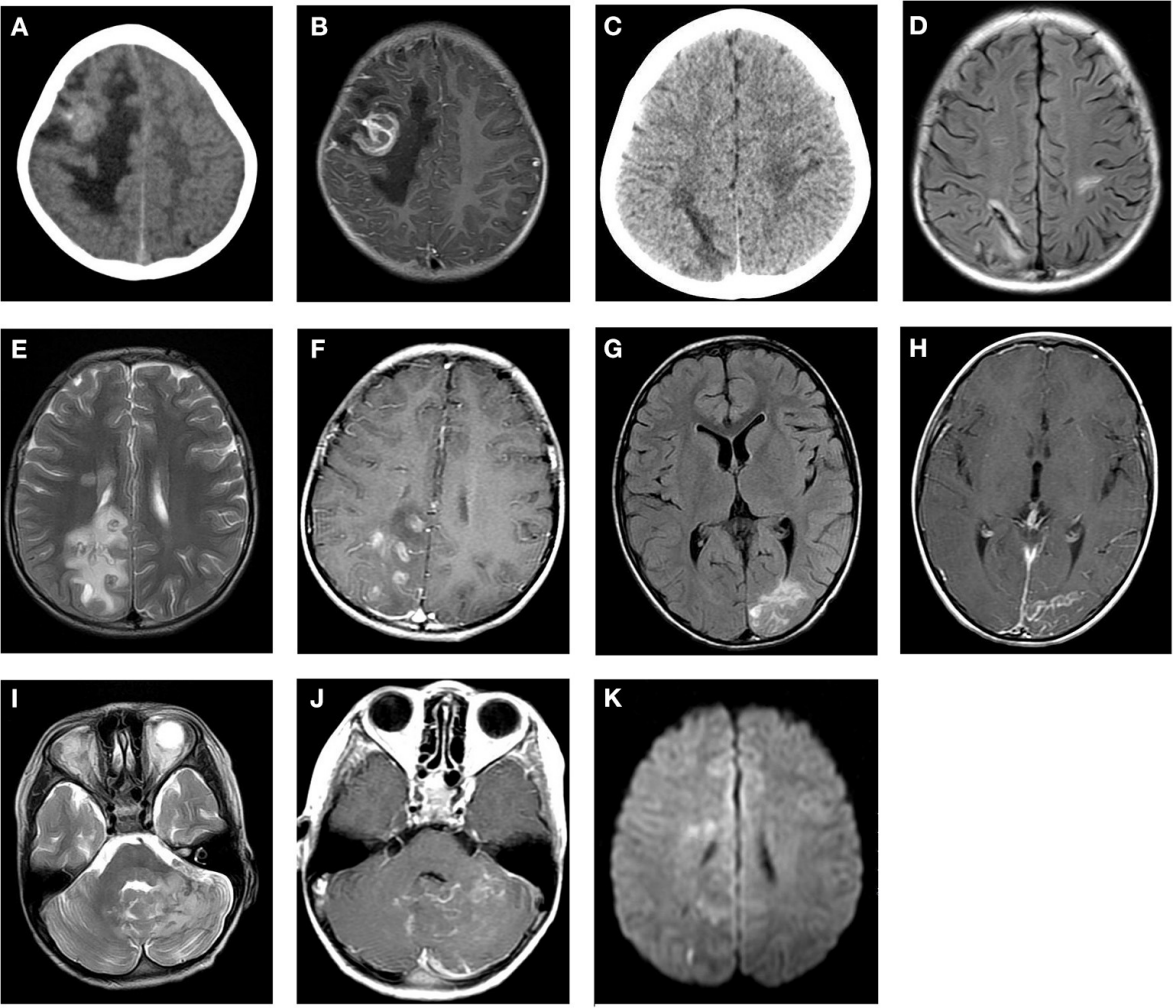
Cranial CT and MRI features of the patients with cerebral paragonimiasis are shown. CT images showed the lesion in the right frontotemporal lobe, which is a mass of equal or slightly high density, surrounded by patchy low-density edema **(A)**. The T1WI enhancement scans showed that the lesion was a ring-like enhancement, surrounded by edema **(B)**. CT images showed a strip-like low-density lesion in the right parietal lobe surrounded by mild edema **(C)**. On the T2/FLAIR images, the lesion was a strip-like low signal surrounded by a high signal, which is the classic “tunnel sign” **(D)**. On the T2WI scans, the lesion in the right parietal occipital lobe was nodular and a circular low or slightly high signal, accompanied by peripheral patchy edema **(E)**. The lesions showed obvious nodular and circular enhancement on the T1WI enhancement scans **(F)**. On the T2/FLAIR images, the left occipital lobe lesions were strip-like and sheet with slightly high signals, and the peripheral edema was not obvious **(G)**. On the T1WI enhancement scans, the lesion showed strip enhancement **(H)**. On the T2WI scans, the left cerebellar hemisphere showed an uneven increased signal, which had nodular and striped mixed signals with unclear boundaries **(I)**. On the T1WI scans, the lesions showed ring-like and strip-like enhancements, and the adjacent meninges were thickened and significantly enhanced **(J)**. On the DWI scans, an equal or slightly high signal is shown **(K)**.

Cranial CT and MRI scans were conducted in the 12 pediatric patients with CP, and the lesions in all 12 cases were located in the cerebral parenchyma, with 10 in the supratentorial area, 2 in the subtentorial area, and involvement of the meninges in three cases (as shown in Table 1). The morphology of the lesions in the CT and MRI was mainly nodular and striated, with clear or indistinct borders and varying degrees of surrounding edema. The lesions showed isodense or slightly hyperdense opacities on the CT scan. The MRI manifested as ring-shaped cystic lesions, with heterogeneous equal or slightly decreased signal intensities on the T_1_WI scans, heterogeneous equal or slightly increased signal intensities on the T_2_WI scans, and equal or slightly increased signal intensities on the DWI scans. In four cases, the cyst wall showed equal T1 and short T2 signals, and in six cases, the characteristic “tunnel sign” and “worm-eaten sign” were visible. The contrast-enhanced MRI showed strip-shaped enhancement in five cases, nodular or ring-shaped enhancement in three cases, linear enhancement in two cases, and uneven enhancement in two cases. The meninges adjacent to the lesions were thickened with significant enhancement in four cases (as demonstrated in Figure 1, Table 2).

**Table 1:** The location distribution of cerebral paragonimiasis.

**Location of the lesion**	**Cases (%)**
The supratentorial area	10 (83)
The subtentorial area	2 (17)

**Table 2:** The MR manifestations of cerebral paragonimiasis.

**The MR manifestations of the lesion**	**Cases**
The cyst wall showed equal T1, short T2	4
The tunnel sign and the worm-eaten sign	6
The linear enhancement	2
The strip enhancement	5
The nodular, ring-shaped enhancement	3
The uneven enhancement	2
The thickening and enhancement of adjacent meninges	4

## Discussion

### Pathogenesis of Cerebral Paragonimiasis

Paragonimiasis is a Zoonotic Parasitic disease mostly caused by eating raw or semi-raw crabs and crickets or drinking raw stream water in endemic areas (6). *Paragonimus* is pathogenic to humans and includes the *Paragonimus westermani* and *Pagumogonimus skrjabini Chen* species. The main pathological features of Paragonimiasis are the formation of sinus and multilocular cysts (7). The pathogenic mechanism is mainly that the metacercariae enter the intestine through the mouth and decapsulate in the small intestine, becoming juvenile. The juveniles enter the abdominal cavity through the diaphragm and enter the lungs where they develop into adult worms. Both adults and larvae of *Paragonimus* have the habit of invasion and migration (1, 8). The mechanism of *Paragonimus* migration to the brain is not very clear. One hypothesis is that immature larvae migrate through the loose connective tissue surrounding the jugular vein or carotid artery, penetrate the meninges, and invade the cerebral tissue. This is consistent with the finding that the distribution of lesions tends to be in the middle cerebral artery and posterior artery. The lesions are often located in the temporal lobe, parietal lobe, and occipital lobe (1). Migration and catabolism of the *Paragonimus* in the cerebral tissue cause an inflammatory response, leading to eosinophil infiltration and granuloma formation with edema of the surrounding cerebral tissue (9, 10). As time progresses, the worm destroys the cerebral tissue, causing necrosis and the formation of partially calcified fragments, which are encapsulated by a cyst wall of granulation and fibrous tissue (8). Meanwhile, eosinophilia can extensively erode the cerebrovascular system and eventually cause rupture of the eroded blood vessels, resulting in various degrees of hemorrhage (10).

### The Clinical Manifestations and Laboratory Examinations

CP is most common in children and adolescents, with a significantly higher incidence in children than in adults, and more common in males than in females (1). Most patients with CP have a slow onset and progressive exacerbation. In a few patients, especially the pediatric patients, the onset may be acute, with the neurological clinical manifestations being mostly headache, dizziness, vomiting, seizure, partial sensory disorders, and other localized brain symptoms; these are often accompanied by respiratory symptoms (11–13). According to the literature, most patients with CP have had respiratory symptoms before the appearance of neurological symptoms, with cough, shortness of breath, and chest pain as the main manifestations (8). In addition, symptoms and manifestations caused by lesions at other sites may develop, and even result in sudden death (6). Due to the lack of specificity, the clinical manifestations of CP are complex and varied, thus it is highly susceptible to being misdiagnosed.

The laboratory examinations for CP include eosinophil counting, intradermal testing for the *Paragonimus* antigen, and ELISA (4, 14). Eosinophil counting is the most simple and easy examination to perform. A large number of eosinophil aggregates can be visible in the cerebral tissue in patients with CP, which can also be accompanied by a large number of plasma cells, neutrophils, lymphocytes, etc. It is an important screening method commonly used in clinical practice. The confirmed diagnosis of CP relies on detection by ELISA of *Paragonimus* antibodies in the serum of the patient (4).

### The Imaging Manifestations and Pathological Analysis

The lesions of CP are often located in the cortical and medullary junction areas of the supratentorial area of the cerebral hemispheres, mostly in the temporal, parietal, and occipital lobes, but can also occur in the subtentorial area (1, 15). The morphology of the lesions in CT and MRI scans is mainly nodular and striated, with varying degrees of surrounding edema. The image appearance varies at different stages of the lesion. For most patients, the most common CT presentation is an isodense or hyperdense lesion, and MRI presents as a ring-shaped cystic lesion (16). With a review of the previous literature, the authors suggested that the “tunnel sign” might be the characteristic imaging manifestation in CP, which appeared on the CT as a strip lesion with decreased intensity and opacity and varying degrees of surrounding edema. On the MRI, the tunnel sign appeared as a decreased intensity signal on the T1WI scans and an increased intensity signal on the T2WI scans, showing a tubular structure with an aperture of 1–3 mm. On the T2/FLAIR images, it appeared as a strip of decreased intensity signal surrounded by increased intensity signal, and it might also appear as a strip or a piece of increased intensity signal. On the T1WI enhanced scans, the lesion appeared as a significantly enhanced tunnel opacity or a significantly enhanced tunnel opacity between two adjacent lesions, which was the migration trajectory of the *Paragonimus* in the brain. The “tunnel sign” reflected the pathological changes that occurred after the penetration of the *Paragonimus* into the cerebral tissue (3, 8, 16). In the present study, the tunnel sign was observed in four cases, and the imaging findings were consistent with those reported in the literature. In addition, the manifestations of ring-shaped, nodular, and linear enhancement on the T1WI enhanced scans were also due to different pathological changes (17). The ring-shaped enhancement was mostly caused by cavities formed by stagnant worms destroying the cerebral tissue, with necrotic and liquefied brain tissue inside, surrounded by a cyst wall composed of granulation and fibrous tissue (7), while the granulomatous hyperplasia and vasculitis in the infiltrative stage might manifest as a nodular enhancement. If the lesion involved the meninges, the adjacent meninges might be thickened with significant enhancement in the T1WI enhanced scan, which might be caused by the invasion of *Paragonimus* migration into the meninges. In the present study, the ring-shaped and nodular enhancements observed in three patients, as well as the meningeal involvement, thickening, and significant enhancement in four patients, were consistent with previous reports in the literature. Two cases manifested as liner enhancement. The different features of enhancement in these cases might be correlated with the different stages of the disease. Intracranial hemorrhage can occur in children with CP, and the hemorrhage signal has a variety of manifestations on MRIs, mainly showing a high T1 signal and an equal, slightly high, or low T2 signal (11, 18). Susceptibility-weighted imaging (SWI) is a sensitive examination for the micro-hemorrhagic foci (19). However, no significant hemorrhage was observed in the cases enrolled in the present study, and no additional SWI sequence scanning was conducted. Therefore, a larger sample size is needed for further investigation.

### Differential Diagnosis

In the diagnosis of cerebral paragonimiasis, attention should be paid to differentiating from cerebral tuberculosis, metastases, brain abscess, glioma, etc: ① Patient with brain tuberculoma mostly has poisoning symptoms or history of tuberculosis, with small tuberculous granuloma in the single brain parenchyma, with the rare occurrence of bleeding and mild peripheral edema, often accompanied by hydrocephalus. ② Patients with brain abscess have local or systemic signs and symptoms of infection, with elevated leukocytes, and one or more thin and luminous annular enhancements on the enhancement scans, with restriction spreading of pus cavity on DWI having the diagnostic value. ③ Brain metastases are common in middle-aged and elderly patients, usually with a history of the primary tumor, and present as multiple scattered nodular or ring-shaped enhancing lesions in the corticomedullary junction area, with unsmooth walls, rare calcifications, and significant perifocal edema disproportionate to the size of the lesion. ④ Gliomas are mostly located in the deep white matter of the brain. There may exist hemorrhage and necrosis within the tumor, which manifest as plaque-like or wreath-like enhancement with obvious space-occupying effect (as shown in Table 3).

**Table 3:** The similarities and differences in the CT and MRI manifestations between other similar lesions and cerebral paragonimiasis.

	**The similarities and differences in images**	
	**The difference**	**The similarity**
Cerebral parenchymal tuberculosis	The tuberculosis is generally small with rare bleeding and mild peripheral edema. If multiple aggregates fuse to form a tuberculous granuloma, there is bead-like or plum-like enhancement, often accompanied by hydrocephalus	With the occurrence of calcification, nodular, ring-like enhancement
Brain abscess	The wall is thin, smooth and regular, with restriction in the pus cavity in DWI	With ring-like enhancement, and obvious peripheral edema
Glioma	Mostly located in the deep white matter of the brain, generally with patchy or wreath-like enhancement, and obvious space-occupying effect	Generally with heterogeneous density/signal, and necrosis and hemorrhage may occur within the lesion, with significant peritumoral edema
Brain metastasis	Mostly with disseminated distribution, and calcification is less common	Prevalent in the corticomedullary junction area, with nodular, circumferential enhancement and significant peri-lesion edema

## Conclusion

CT and MRI scanning can be of great value in the clinical diagnosis of CP. The lesions often manifest as a collection of multiple ring-shaped lesions of different sizes, and the “tunnel sign” with peripheral edema might be the most typical imaging feature in CP. In addition, bilateral cerebral hemisphere involvement, multiple foci in the brain, extensive invasion of adjacent meninges and ventricular walls, and migration of lesions were other noteworthy imaging features. The analysis of imaging features of CP could provide a reference for early diagnosis, but the definitive diagnosis of CP should also be combined with epidemiological history, clinical manifestations, and laboratory examinations. Early diagnosis and timely treatment could reduce the need for surgery and prevent further injury to brain function, which could be critical for patients to achieve a goodprognosis.

## Data Availability Statement

The original contributions presented in the study are included in the article/supplementary material, further inquiries can be directed to the corresponding author/s.

## Ethics Statement

The study was conducted in accordance with the Declaration of Helsinki (as was revised in 2013). The study was approved by Ethics Committee of the Affiliated Hospital of Guizhou Medical University. Written informed consent was obtained from all participants.

## Author Contributions

JD and XL: conception and design of the research. JD and LL: acquisition of data and writing of the manuscript. HF and YY: analysis and interpretation of the data. YL and HY: statistical analysis. XL: obtaining financing. XL and HY: critical revision of the manuscript for intellectual content. All authors read and approved the final draft.

## Funding

This study was funded by the National Natural Science Foundation of China (Grant No. 81960537).

## Conflict of Interest

The authors declare that the research was conducted in the absence of any commercial or financial relationships that could be construed as a potential conflict of interest.

## Publisher's Note

All claims expressed in this article are solely those of the authors and do not necessarily represent those of their affiliated organizations, or those of the publisher, the editors and the reviewers. Any product that may be evaluated in this article, or claim that may be made by its manufacturer, is not guaranteed or endorsed by the publisher.
